# Development of a Claim-Based Hospitalization Appropriateness Screening Tool for Adults in South Korea

**DOI:** 10.34172/ijhpm.9576

**Published:** 2026-06-02

**Authors:** Hyemin Jung, Haibin Bai, Sanghyun Cho, Un-Na Kim, Jin-Seok Lee

**Affiliations:** ^1^Department of Health Policy and Management, Seoul National University College of Medicine, Seoul, Republic of Korea.; ^2^Institute of Health Policy and Management, Seoul National University Medical Research Center, Seoul, Republic of Korea.; ^3^Department of Education and Human Resource Development, Seoul National University Hospital, Seoul, Korea.; ^4^Department of Preventive Medicine, Dankook University Hospital, Cheonan, Republic of Korea.; ^5^Clinical Preventive Medicine Center, Seoul National University Bundang Hospital, Seongnam, Republic of Korea.

**Keywords:** Hospitalization Appropriateness, Inappropriate Admission, Screening Tool, Claim-Based Assessment, South Korea

## Abstract

**Background:**

Unnecessary hospitalizations undermine the efficient use of healthcare resources and may expose patients to preventable safety risks. This study aimed to develop a computerized screening tool for identifying potentially inappropriate adult inpatient admissions using nationwide health insurance claims data in South Korea.

**Methods:**

We conducted a retrospective cross-sectional study using the Health Insurance Review and Assessment Service-National Inpatient Sample (HIRA-NIS) (2017–2019) to develop and assess a claims-based hospitalization appropriateness screening tool (HAST) for adult admissions. Based on the adult hospitalization clinical service criteria from the Appropriateness Evaluation Protocol (AEP), we selected only those criteria directly observable in claims data and operationalized them using standardized electronic data interchange codes. The final tool included nine criteria and episodes meeting none of these criteria were classified as potentially inappropriate admissions (PIAs). HAST was assessed through a single-round of expert review for content validity, mortality-based known-groups comparison, and convergent validity against selected SQLape diagnostic groups. Logistic regression models were applied to identify factors associated with PIA.

**Results:**

The final tool comprised nine screening criteria: (1) receiving general anesthesia, (2) admission to intensive care unit (ICU), (3) receiving hospice care, (4) childbirth, (5) continuous monitoring of vital signs, (6) receiving arterial blood gas analysis (ABGA), (7) application of mechanical ventilator, (8) receiving intravenous injections, and (9) receiving intramuscular injections. Across analytic specifications, 17.5% to 19.2% of admissions were classified as PIAs. PIAs were more frequent among adults aged 20–39 years, females, National Health Insurance (NHI) beneficiaries, and patients admitted to small facilities with fewer than 100 beds.

**Conclusion:**

HAST offers a feasible, reproducible, and scalable method for monitoring the appropriateness of hospital admissions using routinely collected administrative data. It can be used for large-scale surveillance to support health policy evaluation and system-level quality improvement.

## Background

**Key Messages**

**Implications for policy makers**

- The hospitalization appropriateness screening tool (HAST) enables policy-makers to continuously monitor the appropriateness of hospitalization at the national level using existing administrative data, thereby facilitating the detection of temporal trends and institutional variations in potentially inappropriate admission (PIA).
- Policy-makers should interpret HAST findings as indicators of system-level performance rather than as measures of individual hospitals’ performance.
- Higher PIA rates in clinics and small hospitals indicate the need for targeted admission management in facilities with limited gatekeeping capacity.

**Implications for the public**

This study demonstrates that a notable proportion of hospital admissions in South Korea may not require inpatient-level care. Unnecessary hospital stays expose patients to avoidable risks, such as hospital-acquired infections, while consuming limited medical resources that could be better allocated to patients with more severe conditions. The newly developed computerized hospitalization appropriateness screening tool (HAST) system enables continuous and nationwide monitoring of potentially inappropriate admissions (PIAs) in a cost- and time-efficient manner. Based on these monitoring results, health policies can be designed to reduce unnecessary hospitalizations and promote more efficient use of healthcare services.

Inpatient care is a medical service that requires high-cost and high-intensity resources, accounting for one of the largest financial expenditures in healthcare systems.^1-3^ Therefore, assessing and managing inpatient services to reduce unnecessary hospitalizations is crucial when considering efficient allocation of medical resources and the sustainability of national health finances.^4,5^ Moreover, hospital admissions inherently carry risks such as infections, falls, or medication misuse.^6^ Thus, inappropriate admissions may unnecessarily elevate these risks, making it critically important from a patient safety perspective as well.

 The gold standard for assessing the medical necessity of hospitalization is the judgment of a physician who either directly examines the patient or reviews the patient’s medical records.^1^ However, this method can produce biased results due to the subjectivity of evaluators.^1,7-9^ To address this limitation, objective assessment tools such as the Intensity-Severity-Discharge and the Appropriateness Evaluation Protocol (AEP) have been developed.^1,8^ Nevertheless, these tools still determine whether the criteria are met based on reviews of medical records by medical professionals. While expert review enables precise assessment of a patient’s clinical status, it poses limitations for large-scale application due to the evaluators’ expertise requirements and significant time consumption.^10^ However, nationwide analyses are essential to evaluate regional variations in hospitalization appropriateness, institutional patterns, and the effectiveness of policy interventions.

 As an alternative to overcome these limitations, computerized screening tools based on administrative data such as medical claims have recently been proposed. In Italy, indicators measuring the appropriateness of hospitalization are disclosed annually to monitor trends in healthcare utilization.^10^ These indicators are calculated based on computerized discharge summaries including variables such as primary diagnosis, secondary diagnosis, and the Barthel index at the time of admission. SQLape, used in Switzerland, classifies disease groups based on the surgical procedures and diagnoses occurring in hospitalization episodes, and determines the justification for hospitalization based on the characteristics of each disease group.^11^ Such administrative data have the advantage of including nationwide patient information and medical utilization records, enabling large-scale analyses based on consistent criteria.^10,11^ However, studies developing and applying claims-based screening tools for hospitalization appropriateness using nationwide administrative data remain relatively limited.

 All citizens in South Korea are required to enroll in the National Health Insurance Service (NHIS), a single-insurer of South Korea. Additionally, detailed information of medical services actually provided to patients are transferred to NHIS during the medical billing process since reimbursement to providers are mainly based on fee-for-service. Considering these characteristics, applying methodology assessing the appropriateness of hospitalization based on administrative data using the NHIS database would enable stable production of nationally representative results.

 Therefore, this study aimed to develop an adult hospitalization appropriateness screening tool (HAST) based on health insurance claims data and apply it to nationwide data to examine the scale and associated factors of potentially inappropriate admission (PIA). Rather than replacing physician chart review at the individual-patient level, HAST was intended as a transparent, reproducible, and scalable screening tool for population-level surveillance of hospitalization appropriateness. Through this approach, the study sought to generate policy-relevant evidence for admission management and for the evaluation of interventions to improve the efficiency and safety of hospital utilization.

## Methods

 This study was designed to develop and assess a claims-based screening tool for identifying potentially inappropriate hospital admissions at the population level, rather than to replace chart-review-based adjudication of individual admissions.

###  Development of Hospitalization Appropriateness Screening Tool 

 Development of HAST proceeded in four steps. First, we reviewed the adult hospitalization “clinical service criteria” of the AEP.^5^ Because Korean health insurance claims data contain information on billed diagnoses and services actually provided, but not on patients’ clinical condition at the time of admission, only AEP-derived clinical service criteria that could be directly observed in claims data were selected. Through this process, nine candidate services were identified: general anesthesia, admission to intensive care units (ICUs), hospice care, childbirth-related procedures, continuous vital sign monitoring, arterial blood gas analysis (ABGA), mechanical ventilation, intravenous injection, and intramuscular injection. Second, we operationally defined each criterion using these services: (1) receiving general anesthesia, (2) admission to ICU, (3) receiving hospice care, (4) childbirth, (5) continuous monitoring of vital signs, (6) receiving ABGA, (7) application of mechanical ventilator, (8) receiving intravenous injections, and (9) receiving intramuscular injections. In principle, a criterion was considered present if any of the corresponding codes were billed at least once during the hospitalization episode, except the last criterion. For “(9) receiving intramuscular injections,” the criterion was considered satisfied when the average daily number of injections (total number of injections divided by the length of stay), exceeded one. Third, each criterion was matched to claims-observable electronic data interchange codes in Korean for services, procedures, monitoring, or unit-level care, rather than diagnosis-based indications or direct measures of clinical severity (Supplementary file 1, Table S1). Finally, the selected criteria and their operational definitions were reviewed by an expert panel.

###  Building a Dataset for Analysis

 This study used the Health Insurance Review and Assessment Service-National Inpatient Sample (HIRA-NIS) database from 2017 to 2019. HIRA-NIS contains all medical service claims utilized during one year by approximately 10% of hospitalized patients in a given year, sampled through stratification by gender and age group. For the sampled patients, the dataset includes all claims records generated during the study year, including both inpatient and outpatient services. The de-identified dataset was obtained through official request and is publicly accessible to qualified researchers via the HIRA’s open data platform (https://opendata.hira.or.kr). The dataset was accessed between November 4, 2023, and January 30, 2024. The authors did not have access to any personally identifiable information at any stage of the study, including both during and after data analysis.

 Hospitalizations were aggregated into episodes based on claims. Specifically, consecutive inpatient claims from the same institution with a discharge-to-admission gap of 0–1 day were combined into a single hospitalization episode. In contrast, transfers to a different institution were treated as a separate episode. Length of stay (from admission date to discharge date) and total healthcare expenses (including out-of-pocket payments) were calculated for each hospitalization episode. Duplicate information across claims was consolidated at the episode level.

###  Definitions of Variables

 Patient sex was categorized into male and female, while age groups were classified in three groups (20-39, 40-64, ≥65). Insurance types were classified into National Health Insurance (NHI) beneficiaries and medical aid recipients. Medical institutions were classified into five categories based on bed capacity and further categorized by institution type, such as clinics, hospitals, general hospitals, and tertiary hospitals.

 Each hospitalization episode was classified as appropriate if at least one of the nine HAST criteria was observed during the episode. Episodes in which none of the criteria were observed were classified as PIAs.

###  Assessment of the Screening Tool

 Because this study was based on de-identified nationwide claims data, no clinical gold standard based on direct patient examination or medical record review was available for comparison. Therefore, HAST was assessed in three ways: content validity, mortality-based known-groups comparison, and convergent validity.^12,13^

- Content validity: We conducted a single-round expert review of the selected criteria and their claims-based operational definitions. The panel included four physicians from internal medicine, surgery, and preventive medicine, and 16 nurses, including two with intensive care unit experience. Experts were selected based on their clinical backgrounds and relevance to inpatient care and hospitalization review. Because the review was intended to assess the face and content validity of the proposed claims-based criteria, no formal cutoff was applied for criterion removal.
- Mortality-based known-groups comparison: We compared inpatient mortality rates between the appropriate and PIA groups, using mortality recorded in the discharge disposition field as a proxy outcome reflecting clinical severity.
- Convergent validity: We compared HAST classifications with selected SQLape diagnostic groups classified as ‘almost never’ requiring hospitalization. Among the 88 diagnostic groups classified in almost-never diagnostic groups, there were 42 disease groups that Korean specialists considered inappropriate even in the Korean context. Relevant disease groups and Korean Classification of Diseases, 8th Revision (KCD-8) codes are listed in Table S2.

###  Statistical Analysis and Ethical Considerations

 The results were presented primarily using descriptive statistics. PIA rates, distributions according to patient and institutional characteristics, and annual trends were calculated. Factors associated with PIA were analysed using logistic regression models. Statistical analyses were performed using Microsoft Excel 2016 and SAS Enterprise Guide 7.1 (SAS Institute Inc., Cary, NC, USA).

 This study was approved and granted exemption from review by the Institutional Review Board of the corresponding author’s institution. Because the data used were fully de-identified administrative claims, the requirement for informed consent was waived.

## Results

###  General Characteristics

 A total of 2 627 375 hospitalization episodes extracted from the HIRA-NIS between 2017 and 2019 were included in the analysis. Table 1 presents the number of hospitalization episodes per year, along with the duration of hospitalization and medical expenditure statistics, while detailed patient demographics and institutional characteristics are provided in Table S3.

**Table 1:** Duration of Hospitalization and Medical Expenditure Per Episode (2017–2019)

		**2017 (N = 864 040)**	**2018 (N = 882 441)**	**2019 (N = 880 894)**
Duration of hospitalization (days)	Mean	8.3	8.1	7.9
	SD	18.0	17.7	18.1
	Max	395.0	365.0	433.0
	Median	4.0	4.0	4.0
Medical expenditure (KRW)	Mean	2 199 927	2 401 507	2 599 132
	SD	4 604 435	5 106 332	5 585 585
	Max	361 960 000	356 584 000	437 158 000
	Median	1 060 000	1 150 000	1 246 000

Abbreviations: SD, standard deviation; KRW, South Korean won.

 Across 2017-2019, the duration of hospitalization showed only modest year-to-year variation, while the median length of stay remained 4 days. The mean expenditure per hospitalization was approximately 2.20 million KRW (South Korean won) in 2017 to nearly 2.60 million KRW in 2019, while the median expenditure was 1.06 million KRW and 1.25 million KRW, respectively.

###  Development of Hospitalization Appropriateness Screening Tool 

 The HAST was applied to 2 627 375 hospitalization episodes from 2017 to 2019. Overall, 81.8% of episodes met at least one of the nine criteria and were classified as appropriate admissions. On average, each episode satisfied 1.42 criteria, with the majority fulfilling just one criterion. Among individual criteria, “(8) receiving intravenous injections” was most frequently met (80.8%), followed by “(6) receiving ABGA” (23.9%) and “(5) continuous monitoring of vital signs” (13.5%). High-intensity medical procedures such as “application of mechanical ventilator” (1.2%) or “admission to ICU” (3.6%) showed relatively lower frequencies (Table 2).

**Table 2:** Proportion of Hospitalization Episodes Meeting Each Screening Criterion in the Hospitalization Appropriateness Screening Tool (2017–2019)

**Screening Criterion**	**Total (N=2627375)**	**2017 (N=864040)**	**2018 (N=882441)**	**2019 (N=880894)**
**Overall**	**81.8%**	**82.5%**	**82.2%**	**80.8%**
1. Receiving general anesthesia	10.2%	10.2%	10.2%	10.2%
2. Admission to ICU	3.6%	3.4%	3.7%	3.7%
3. Receiving hospice care	0.0%	0.0%	0.0%	0.0%
4. Childbirth	2.1%	2.5%	2.1%	1.8%
5. Continuous monitoring of vital signs	13.8%	13.5%	14.0%	13.9%
6. ABGA	23.9%	24.3%	23.9%	23.5%
7. Application of mechanical ventilator	1.2%	1.1%	1.2%	1.1%
8. Receiving intravenous injections	80.8%	81.4%	81.2%	79.9%
9. Receiving intramuscular injections	5.8%	6.5%	5.8%	5.3%

Abbreviations: ICU, intensive care unit; ABGA, arterial blood gas analysis.

###  Assessment of Hospitalization Appropriateness Screening Tool 

 Regarding content validity, among the nine items evaluated by a panel of 20 experts, five showed positive content validity ratio (CVR), one item scored zero, and four items had negative CVR values, with an overall mean CVR of 0.12. For mortality-based known-groups comparison, the inpatient mortality rate among admissions classified as PIA was 0.12%, significantly lower than the mortality rate of 1.42% observed in appropriate admissions (*P* < .001). Convergent validity was assessed by comparison with the SQLape tool developed in Switzerland. Episodes considered appropriate by both tools accounted for 68.1%, while those considered inappropriate by both tools were 1.3%, yielding a Cohen’s kappa coefficient of 0.75.

###  Overall Potentially Inappropriate Admission Rates by Year

 Across 2017–2019, PIA rates showed modest year-to-year differences. Annual PIA rates were 17.5% in 2017, 17.8% in 2018, and 19.2% in 2019 (Table 3).

**Table 3:** Overall Potentially Inappropriate Admission Rates by Year (2017–2019)

		**2017 (N=864040)**	**2018 (N=882441)**	**2019 (N=880894)**
**Overall**		17.5 (17.5–17.6)	17.8 (17.8–17.9)	19.2 (19.1–19.3)
Age group (y)	20-39	24.1 (23.9–24.3)	24.7 (24.5–24.9)	25.7 (25.5–25.9)
	40-64	15.7 (15.6–15.8)	16.2 (16.1–16.3)	17.7 (17.6–17.8)
	≥65	16.0 (15.9–16.1)	16.1 (16.0–16.3)	17.5 (17.4–17.7)
Sex	Male	16.3 (16.2–16.4)	16.7 (16.6–16.8)	18.2 (18.1–18.3)
	Female	18.6 (18.4–18.7)	18.8 (18.7–18.9)	20.0 (19.9–20.1)
Insurance	NHI	18.3 (18.2–18.4)	18.6 (18.5–18.7)	20.0 (19.9–20.1)
	MA	7.9 (7.7–8.1)	7.4 (7.2–7.6)	7.7 (7.5–7.9)
Region of medical institution	Capital regions	18.0 (17.9–18.1)	18.9 (18.7–19.0)	20.7 (20.5–20.8)
	Metropolitan cities	18.6 (18.4–18.7)	18.7 (18.6–18.9)	19.8 (19.7–20.0)
	Other regions	15.9 (15.8–16.0)	15.4 (15.3–15.6)	16.3 (16.1–16.4)
Type of medical institution	Clinic	54.0 (53.7–54.3)	56.8 (56.5–57.1)	60.6 (60.3–60.8)
	Hospital	18.9 (18.8–19.1)	18.9 (18.7–19.0)	19.5 (19.4–19.7)
	General hospital	9.1 (9.0–9.2)	8.7 (8.6–8.8)	8.8 (8.7–8.9)
	Tertiary hospital	7.1 (7.0–7.2)	7.8 (7.7–7.9)	8.7 (8.6–8.9)
Capacity of medical institution	No bed	95.1 (94.8–95.3)	85.5 (85.1–85.9)	94.8 (94.5–95.0)
	1-50 beds	42.4 (42.1–42.6)	43.6 (43.4–43.9)	46.5 (46.2–46.7)
	51-100 beds	18.7 (18.5–19.0)	18.8 (18.6–19.1)	18.4 (18.1–18.7)
	101-300 beds	11.2 (11.1–11.4)	10.6 (10.4–10.7)	10.3 (10.2–10.5)
	301-500 beds	10.3 (10.1–10.5)	10.2 (10.0–10.4)	11.0 (10.8–11.3)
	501-1000 beds	8.4 (8.3–8.5)	8.4 (8.3–8.5)	8.7 (8.6–8.8)
	≥1001 beds	7.4 (7.2–7.5)	8.2 (8.0–8.3)	9.4 (9.2–9.5)
Type of entry	Transferred and ER	7.0 (6.9–7.1)	6.9 (6.8–7.0)	7.3 (7.2–7.4)
	Outpatient clinic	20.8 (20.7–20.9)	21.3 (21.2–21.4)	22.8 (22.7–22.9)
Hospitalization outcome	Discharged	16.7 (16.6–16.8)	16.9 (16.8–16.9)	17.9 (17.8–18.0)
	Continued	24.9 (24.6–25.1)	26.0 (25.8–26.3)	29.2 (28.9–29.5)
	Deceased	1.6 (1.3–1.8)	1.9 (1.6–2.1)	2.0 (1.8–2.3)

Abbreviations: NHI, National Health Insurance; MA, medical aid; ER, emergency room.

 The PIA rate was higher in females than males, and highest in the youngest age group. According to insurance type, the rate was relatively lower among medical aid recipients than NHI beneficiaries. Notable differences in PIA rates were observed across types and sizes of medical institutions. Clinics exhibited the highest PIA rates, with a decreasing trend observed in higher-tier institutions such as hospitals, general hospitals, and tertiary hospitals. A similar pattern was found with respect to bed capacity: institutions with fewer beds tended to have higher PIA rates, with particularly elevated rates seen in facilities with 100 beds or fewer.

###  Factors Associated With Potentially Inappropriate Admission

 To identify factors associated with PIA, stepwise logistic regression analysis was conducted using a combined dataset from 2017 to 2019, with PIA status as a dependent variable (Table 4).

**Table 4:** Univariate and Multivariate Logistic Regression Models Predicting Potentially Inappropriate Admission (N = 2 627 375)

	**Variables**	**Univariate**		**Multivariate**	
		**cOR**	**95% CI**	**aOR**	**95% CI**
Age group	20-39	4.69	4.64-4.74	2.99	2.95-3.02
	40-64	2.09	2.07-2.11	1.63	1.62-1.65
	≥65	Reference		Reference	
Sex	Male	Reference		Reference	
	Female	1.21	1.20-1.22	1.01	1.00-1.02
Insurance	NHI	2.55	2.50-2.61	1.52	1.49-1.56
	MA	Reference		Reference	
Region of medical institution	Capital regions	1.37	1.35-1.38	1.42	1.41-1.44
	Metropolitan cities	1.31	1.30-1.33	1.39	1.37-1.40
	Other regions	Reference		Reference	
Type of medical institution	Clinic	9.24	9.12-9.36	2.88	2.79-2.97
	Hospital	2.77	2.74-2.81	1.76	1.71-1.80
	General hospital	1.32	1.31-1.34	1.35	1.32-1.38
	Tertiary hospital	Reference		Reference	
Capacity of medical institution	No bed	7.89	7.56-8.24	2.57	2.44-2.71
	1-50 beds	8.07	7.94-8.19	2.51	2.43-2.59
	51-100 beds	3.07	3.01-3.12	1.50	1.45-1.55
	101-300 beds	1.48	1.45-1.50	1.01	0.99-1.04
	301-500 beds	1.39	1.36-1.42	1.14	1.11-1.17
	501-1000 beds	1.10	1.09-1.12	1.07	1.04-1.09
	≥1001 beds	Reference		Reference	
Type of entry	Transferred and ER	Reference		Reference	
	Outpatient clinic	2.68	2.65-2.71	1.49	1.47-1.50
Hospitalization outcome	Discharged	8.07	7.42-8.78	2.90	2.66-3.15
	Continued	7.93	7.29-8.64	3.81	3.50-4.15
	Deceased	Reference		Reference	

Abbreviations: cOR, crude odds ratio; aOR, adjusted odds ratio; CI, confidence interval; NHI, National Health Insurance; MA, medical aid; ER, emergency room.

 In univariate analysis, age, sex, insurance type, location of the medical institution, type of medical institution, institution capacity (number of beds), admission route, and admission outcomes were all significantly associated with PIA (*P* < .01).

 Multivariate logistic regression, including all statistically significant variables from the univariate analysis, indicated that all variables except sex remained significantly associated with PIA. Key variables showing more than a twofold difference in PIA probability included: patients aged 20–39, who had a 2.99-fold (95% CI: 2.95–3.02) higher probability of PIA compared to those aged ≥65; admissions to clinic-level institutions, with a 2.88-fold (95% CI: 2.79–2.97) higher PIA probability compared to tertiary hospitals; and patients admitted to institutions without beds or with 1–50 beds, with respective probabilities of 2.57-fold (95% CI: 2.44–2.71) and 2.51-fold (95% CI: 2.43–2.59) higher compared to institutions with ≥1001 beds. Lastly, patients remaining hospitalized or transferred to other institutions had a 3.81-fold (95% CI: 3.50–4.15) higher probability of PIA compared to deceased patients.

## Discussion

 This study developed HAST, a computerized screening tool assessing the appropriateness of hospitalization, and applied it to national-level claim data. Medical procedures actually provided to patients are unlikely to be omitted from claims, as unclaimed services are not imbursed under fee-for-service payment system. In this sense, claims-observable services provide a useful basis for standardized, reproducible, and large-scale assessment of hospitalization appropriateness. At the same time, some services may be routinely or excessively used in inpatient practice under a fee-for-service system. When such services are treated as indicators of admission appropriateness, PIA may be underestimated. This possibility is illustrated by the high frequency of intravenous and intramuscular injections in our data. Future refinements of HAST may therefore need to incorporate more clinically specific information, such as the type of administered medication and the need for close inpatient monitoring, in order to improve the specificity of the tool.

 The validity of the HAST was evaluated from three perspectives. For content validity, more than half of the respondents agreed positively (CVR > 0) on six of the nine items, excluding ABGA, intravenous injections, and intramuscular injections. However, even for these three criteria, experts generally acknowledged their usefulness but recommended clearer specifications or frequency restrictions for inclusion. Regarding mortality-based known-groups comparison, the inpatient mortality rate was lower among patients classified as PIA (0.12%) than among appropriate admissions (1.42%), supporting the hypothesis that patients classified as PIA generally had lower clinical severity. The comparison with the SQLape system showed substantial agreement (Cohen’s kappa = 0.75), supporting the convergent validity of HAST as a claims-based screening tool. Because the two tools are based on different classification logics, this finding should be interpreted as evidence of convergence rather than equivalence. Taken together, this finding supports the use of HAST as a feasible and reproducible screening tool for large-scale analyses based on administrative data.

 Approximately 17.5%-19.2% of total hospital admissions were classified as PIA, aligning reasonably with prior domestic and international studies.^1,3,9-11,14-18^ For example, Switzerland’s SQLape criteria indicated a 6.5% rate of unjustified stays,^11^ while recent Italian study suggested inadequate occupancy rate of acute care beds ranging from a minimum of 6% to a maximum of 28%.^10^ Although the observed proportion of PIA was similar or lower than these countries, it is unlikely that the actual prevalence of PIA in South Korea is low. This discrepancy likely arose because HAST is primarily based on whether specific procedures or treatments were performed during hospitalization. If such procedures are themselves overutilized, their presence could artificially lower the measured rate of PIA.

 Among patient sociodemographic characteristics, age showed the strongest association with PIA rates. Our finding of lower PIA rates among older adults is broadly consistent with prior literature suggesting that older patients are more likely to have greater clinical severity and comorbidity burden at the time of admission.^19^ Even when elderly patients with relatively mild symptoms are hospitalized for observation or monitoring, such hospitalization is considered appropriate because continuous vital sign monitoring services are provided and billed.

 It is noteworthy that medical aid recipients had lower PIA rates than health insurance subscribers, contrary to common assumptions that lower co-payments would result in more inappropriate admissions.^20^ This suggests the lower PIA rates among medical aid recipients may reflect greater medical need rather than overuse, given their higher burden of illness.^21^

 Some differences in PIA rates were observed based on the size and type of healthcare institution. Admissions recorded in facilities classified as having no beds may reflect day-bed admissions, which are treated as inpatient care for billing purposes when chemotherapy, procedures, or surgery are followed by at least 6 hours of observation; accordingly, these episodes may differ from conventional ward-based admissions and may be more likely to be classified as PIA under HAST. Clinics and small hospitals (≤50 beds) showed relatively high PIA rates, whereas large general hospitals (>200 beds) demonstrated lower rates. The difference may reflect “supplier-induced demand” commonly observed in small hospitals or clinics with low bed occupancy rates. In a Delphi study from Spain, “tactical use of beds”—defined as practices like filling beds to manage a service or ward, or delaying patient discharge until another patient is admitted—was identified as a cause of inappropriate admissions.^7^ Similarly, an Australian study noted that 70% of acute hospital bed-days were occupied by patients awaiting residential care, rehabilitation services, or home support.^22^ Thus, higher rates of PIA in smaller hospitals may reflect a combination of provider-induced demand, and the strategic management of hospital beds.

 This study has several limitations. First, because the analysis was based on administrative claims data, admissions composed only of non-covered services may not have been fully captured. However, given that non-covered services in South Korea are generally accompanied by covered procedures^23^ this issue is likely limited. Second, HAST does not fully capture the clinical nuances obtainable from chart reviews or bedside assessments. Therefore, this tool is valuable primarily as a screening instrument for identifying “potential” inappropriateness rather than as a definitive method for patient-level judgment. Third, the use of annual sample data may have led to underestimation of long-term hospitalizations extending beyond one year. Fourth, this study could not assess the appropriateness of individual hospital days because claims data record services at the episode level rather than on the exact day of service during hospitalization. Future studies utilizing more detailed clinical or time-stamped data may be able to evaluate potentially inappropriate hospital days in addition to admission-level appropriateness. Finally, the developed tool applies exclusively to general adult admissions, and separate criteria development is required for specialized areas such as pediatric, psychiatric, or long-term care hospitals.

 Despite these limitations, this study demonstrates the feasibility of generating and continuously monitoring national PIAs using the computerized claims-based screening tool. The value of HAST lies not in replacing physician judgment or chart-review-based assessment, but in providing a transparent, reproducible, and scalable framework that can be applied repeatedly to nationwide administrative data. In this respect, HAST may complement existing approaches by supporting system-level surveillance of temporal trends, institutional variation, and policy-relevant patterns in hospitalization appropriateness. However, careful implementation is essential to avoid compromising the quality of healthcare services. Further research and ongoing validation to refine the criteria are essential to facilitate practical application of this tool in the real-world setting.

## Conclusion

 This study developed a claims-based HAST for identifying potentially inappropriate adult hospital admissions in South Korea. HAST offers a feasible, reproducible, and scalable approach for monitoring hospitalization patterns using routinely collected administrative data. Although it does not replace physician chart review for individual-level adjudication, it may serve as a useful tool for system-level surveillance of temporal trends, institutional variation, and policy-relevant patterns in PIA.

## Disclosure of artificial intelligence (AI) use

 ChatGPT (OpenAI) was used solely to assist with language editing of the manuscript, and all of the intellectual content, interpretations, and final revisions were made by the authors.

## Ethical issues

 This study was approved by the Institutional Review Board of Seoul National University Hospital (IRB No.: E-2306-064-1438), and the requirement for informed consent was waived because de-identified NHI claims data were used.

## Conflicts of interest

 Authors declare that they have no conflicts of interest.

## Disclaimer

 The views expressed in this article are solely those of the authors and do not necessarily reflect the views or policies of Seoul National University or the Health Insurance Review and Assessment Service.

## Supplementary files


Supplementary file 1 contains Tables S1-S3.
